# Influence of Cage Design on Radiological and Clinical Outcomes in Dorsal Lumbar Spinal Fusions: A Comparison of Lordotic and Non‐Lordotic Cages

**DOI:** 10.1111/os.12872

**Published:** 2021-03-24

**Authors:** Christian Walter, Tobias Baumgärtner, Dominik Trappe, Sandra Frantz, Lisanne Exner, Moritz Mederake

**Affiliations:** ^1^ University Hospital Tübingen Tübingen Germany

**Keywords:** Cage, Interbody fusion, Lumbar lordosis, Sagittal alignment, Spine surgery

## Abstract

**Objectives:**

To evaluate the comparison between lordotic and non‐lordotic transforaminal lumbar interbody fusion (TLIF) cages in degenerative lumbar spine surgery and analyze radiological as well as clinical outcome parameters in long‐term follow up.

**Methods:**

In a retrospective study design, we compared 37 patients with non‐lordotic cage (NL‐group) and 40 with a 5° lordotic cage (L‐group) implanted mono‐ or bi‐segmental in TLIF‐technique from 2013 to 2016 and analyzed radiological parameters of pre‐ and postoperative (Lumbar lordosis (LL), segmental lordosis (SL), and pelvic tilt (PT), as well as clinical parameters in a follow‐up physical examination using the Oswestry disability index (ODI), Roland–Morris Score (RMS), and visual analog scale (VAS).

**Results:**

Surgery was mainly performed in lower lumbar spine with a peak in L4/5 (mono‐segmental) and L4 to S1 (bi‐segmental), long‐term follow‐up was on average 4 years postoperative. According to the literature, we found significantly better results in radiological outcome in the L‐group compared to the NL‐group: LL increased 6° in L‐group (51° preoperative to 57° postoperative) and decreased 1° in NL‐group (50° to 49° (*P* < 0.001). Regarding SL, we found an increase of 5° in L‐group (13° to 18°) and no difference in NL‐group (15°)(*P* < 0.001). In PT, we found a clear benefit with a decrease of 2° in L‐group (21° to 19°) and no difference in NL‐group (*P* = 0.008).

In direct group comparison, ODI in NL‐group was 23% *vs* 28% in L‐group (*P* = 0.25), RMS in NL‐group was 8 points *vs* 9 points in L‐group (*P* = 0.48), and VAS was in NL‐group 2.7 *vs* 3.2 in L‐group (*P* = 0.27) without significant differences.

However, the clinical outcome in multivariate analysis indicated a significant multivariate influence across ODI and RMS of BMI (Wilks λ = 0.57, F [4, 44] = 3.61, *P* = 0.012) and preoperative SS (Wilks λ = 0.66, F [4, 44] = 2.54, *P* = 0.048). Age, gender, cage type and postoperative PT had no significant influence (*P* > 0.05). Intraoperatively, we saw three dura injuries that could be sutured without problems and had no consequences for the patient. In the follow‐up, we did not find any material‐related problems, such as broken screws or cage loosening, also no pseudarthrosis.

**Conclusion:**

In conclusion, we think it's not cage design but other influenceable factors such as correct indication and adequate decompression that lead to surgical success and the minimal difference in the LL therefore seemed to be of subordinate importance.

## Graphical abstract

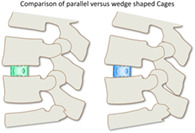

## Introduction

In recent years, interbody fusion techniques in combination with spinal instrumentation have become increasingly popular in the surgical therapy of degenerative spine diseases. Despite surgical advances, pseudarthrosis and instrumentation failure are still common problems, especially in the lumbosacral junction^1^, ^2^. In order to address this problem, one goal of surgical interventions is to increase the rigidity of the construct. In many cases, an interbody fusion device in addition to posterior screws (360° fusion) is used as a further stabilization option^3^.

Concerning interbody fusion techniques, the use of stand‐alone bone grafts was largely replaced by titanium or polyetheretherketone (PEEK) cages in combination with cancellous bone^4^, ^5^, ^6^. The cages offer many advantages. First, the cage provides additional anterior column support resulting in higher stiffness of interbody fusion, compared with posterior fusion only in biomechanical testing^7^. Secondly, depending on the cage type, a large surface area for fusion and an indirect decompression of the neuroforamen is attainable^8^. Finally, the surgeon has the ability to restore spinal balance either by sagittal alignment using a wedge‐shaped (lordotic) cage due to geometry or segmental coronary alignment by adapting posterior rod compression to the deformity^9^. Therefore, the implantation of a cage is mandatory for many spine surgeons when performing dorsal lumbar spondylodesis.

When inserting the cage, various options for surgical approach are available. Firstly, the anterior lumbar interbody fusion (ALIF) with transperitoneal or retroperitoneal approach to the lumbar spine is the oldest procedure^10^. Secondly, the posterior lumbar interbody fusion (PLIF) technique involves a cage or bone chip being inserted posterior on both sides of the dura into the disc space and therefore the dura is mobilized medially. This technique dates back to Cloward in the 1980s and is still widely used today^11^, ^12^.

We used in the present study the transforaminal approach (TLIF, transforaminal lumbar interbody fusion), which was developed by Harms in the 1990s^13^. The principle of this approach is the expansion of the intervertebral space, as well as secure ventral support and fusion through autogenous bone and the one‐sided transforaminal inserted cage. The segment stability is then restored by converting the distraction force into compression force. The technique has been further developed through the construction of banana‐shaped cages and is currently the standard in many clinics^14^.

Worldwide, PLIF and TLIF technologies are currently the most commonly used techniques for 360° fusion. However, compared to the PLIF technique, the neural structures have to be mobilized less with the TLIF technique, which leads to a lower complication rate (dural lesions, nerve root damage)^15^. Another argument is the unilateral approach, which is only possible with the TLIF technique, while the PLIF technique always requires a bilateral cage insertion^16^. Therefore, from the authors' perspective, the TLIF technique is currently the preferred method.

However, the posterior approach denies direct access to the intervertebral disc compartment, resulting in smaller cages compared to the ALIF^17^. In order to attain, larger cages and reduced access morbidity, laparoscopic ALIF techniques and the minimally invasive XLIF technique was developed, achieving reduced access morbidity and shortening the operating time compared to the open ALIF technique^18^, ^19^.

Another argument, in addition to the approach, is the geometry of the cage. So improved lumbar lordosis (LL) could be showed in previous studies, using lordotic cages compared to non‐lordotic (rectangular) cages, regardless of approach. Improved radiological parameters concerning the sagittal balance could be shown for the PLIF technique and the XLIF technique^20^, ^21^. This is an important point, because well‐balanced lordosis of lumbar spine enables efficient muscle work when standing upright and loss of distal LL is often responsible for sagittal imbalance^20^, ^22^. Despite an enormous interest in lumbar lordosis in various scientific studies and the preoccupation of surgeons, therapists, and patients with this topic, the optimal lordotic range remains unidentified^23^, ^24^, ^25^. However, the correlation between LL and lumbar back pain (LBP) is largely undisputed. So, epidemiological studies could demonstrate a significantly reduced LL in patients with LPB compared to a control group.

Despite this known correlation between LL and LBP and the knowledge about improved LL in lordotic cages, it is unknown whether the use of lordotic cages leads to improved clinical outcome compared to non‐lordotic cages, especially in the long‐term follow‐up.

The purpose of the study was to: (i) confirm the improvement in lumbar lordosis (LL), segmental lordosis (SL), and pelvic tilt (PT) described in the literature of our patient population using lordotic TLIF cages; (ii) investigate if the expected radiological improvement leads to a superior clinical outcome using lordotic cages compared with non‐lordotic cages in the follow‐up by detecting Oswestry Disability Index (ODI), Roland–Morris Score (RMS), and Visual Analog Scale (VAS); and (iii) evaluate different influencing factors on postoperative ODI and RMS.

To answer this question, we clinically and radiologically examined 80 patients treated with mono‐ or bi‐segmental TLIF cage.

## Patients and Methods

### Inclusion and Exclusion criteria

**Fig. 1:**
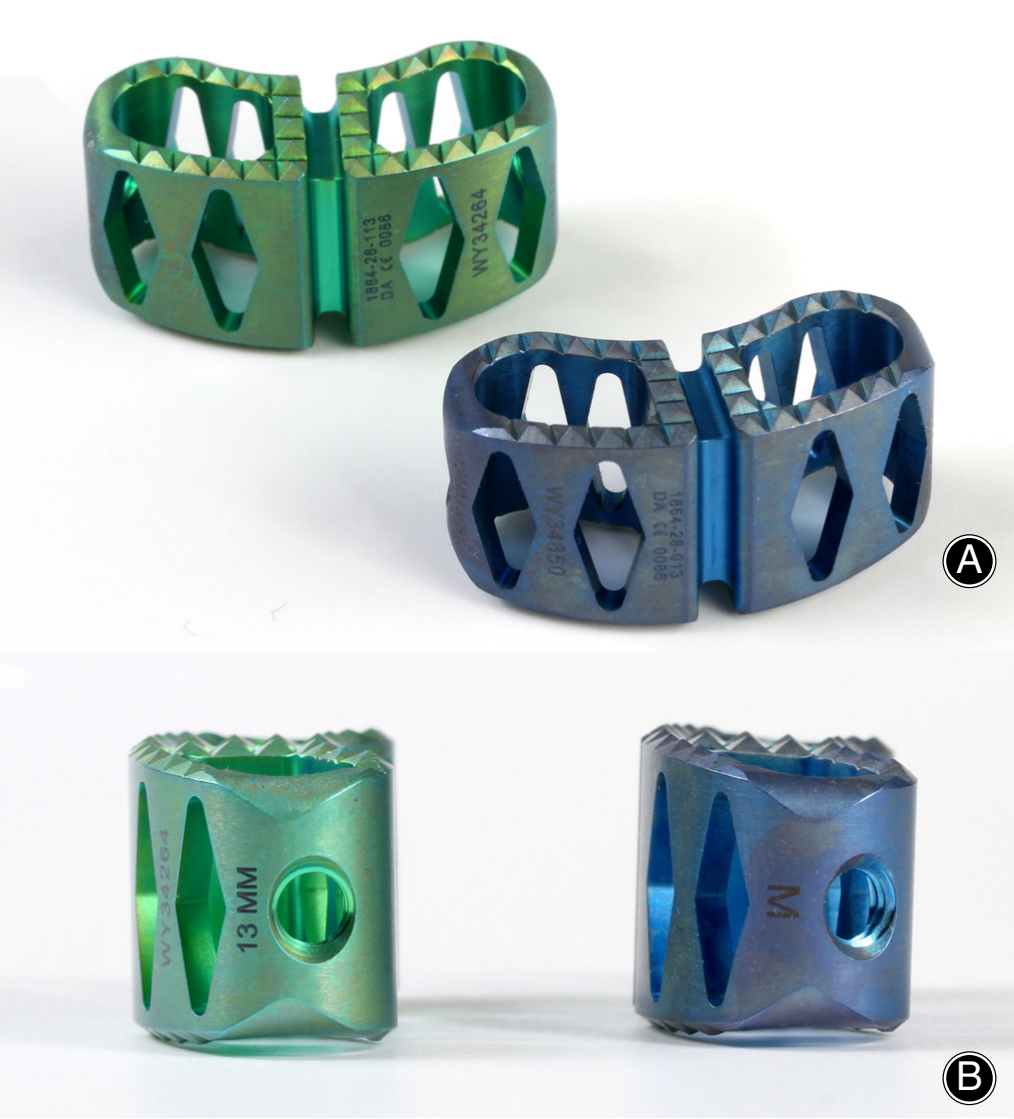
Photos of the two cage types. Green: Non‐lordotic cage, Blue: Lordotic cage (A) Anterior view of the titanium cage used in our study (Devex Cage, Depuy Synthes, Raynham, Massachusetts, USA) with spikes for better anchoring and a large cavity for the attachment of autologous bone. (B) Sagittal view with 0° lordosis (green cage) and 5° lordosis (blue cage) (See figure_2.jpg).

The inclusion criteria were: (i) adults, 30 years or older with degenerative spinal disease underwent mono‐ or bi‐segmental spinal fusion with TLIF on the lumbar spine; (ii) minimum of 7 points on the visual analog scale (VAS) preoperative; (iii) written consent (after clarification) to the study; (iv) intervention was spinal fusion performed with lordotic cage design (see Fig. 1); (v) comparison group became non‐lordotic cage design; (vi) outcome measures were radiological parameters (LL, SL, PT) and clinical scores (ODI, RMS, VAS); and (vii) a retrospective study with prospective follow‐up examination of the patients. Exclusion criteria were as follows: (i) patients with spondylolisthesis more than Meyerding degree I; (ii) more than two instrumented levels or previous spinal fusion; and (iii) previous documentation of osteopenia, osteoporosis, or osteomalacia to a degree that spinal instrumentation would be contraindicated, immunosuppressive disorder, or history of substance abuse (drugs or alcohol).

### Patient Data

**Fig. 2:**
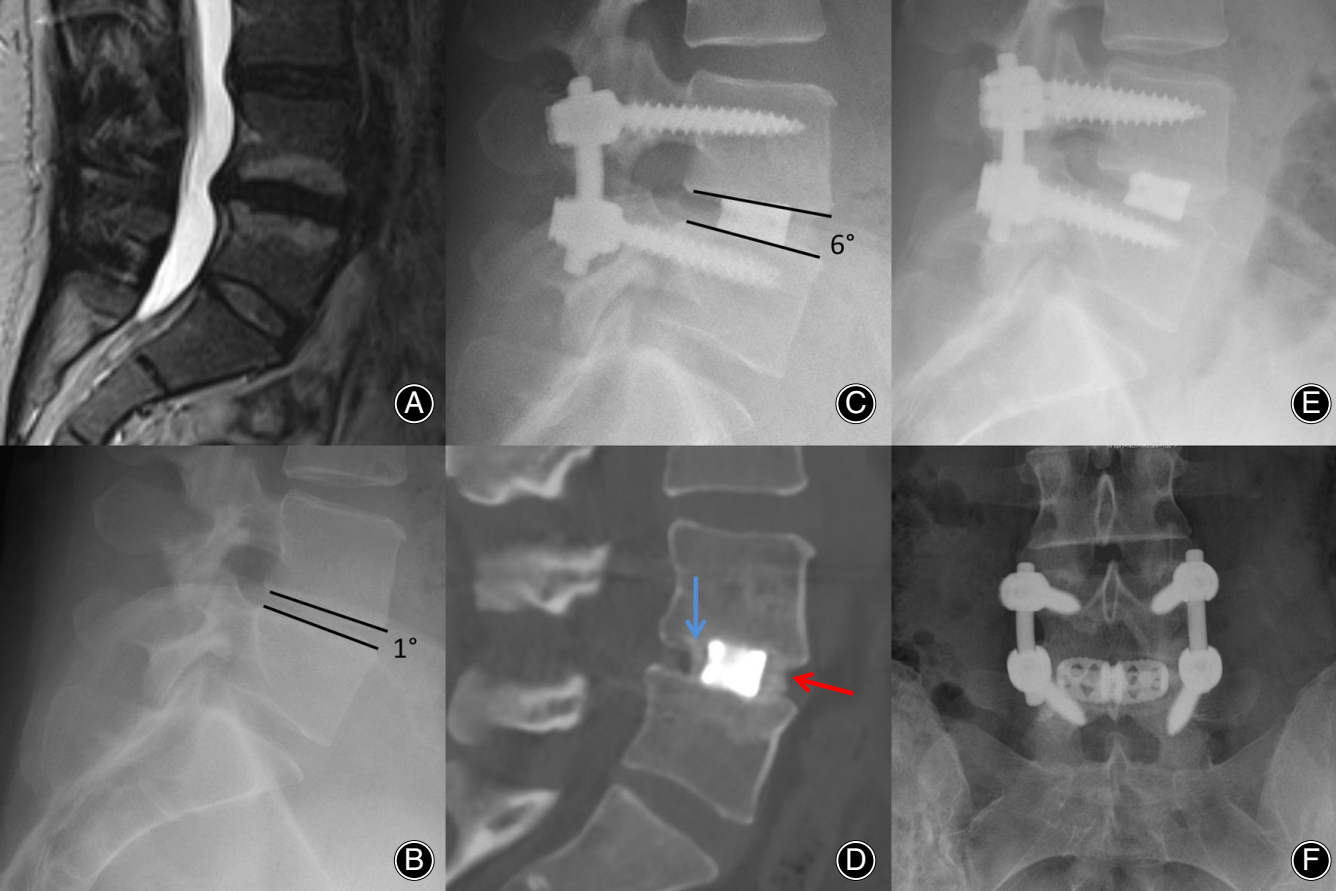
(A) Preoperative MRI Scan of a 36‐year old woman with severe osteochondrosis L 4/5 and disc herniation. Clinically, she complains of therapy‐resistant back pain and radiation to her left leg. (B) Preoperative lateral X‐ray shows a segmental lordosis L 4/5 of 1°. (C) The follow‐up 5 days after surgery with mono‐segmental fusion and implantation of a lordotic cage shows an improved segmental lordosis of 6° (increase of 5°). (D) The CT control after 1 year shows a stable bony fusion posterior to the cage (blue arrow) and anterior to the cage (red arrow). (E, F) The final X‐ray control 2 years after surgery demonstrates a significantly increasing bony fusion compared to (C) and (D).

**Fig. 3:**
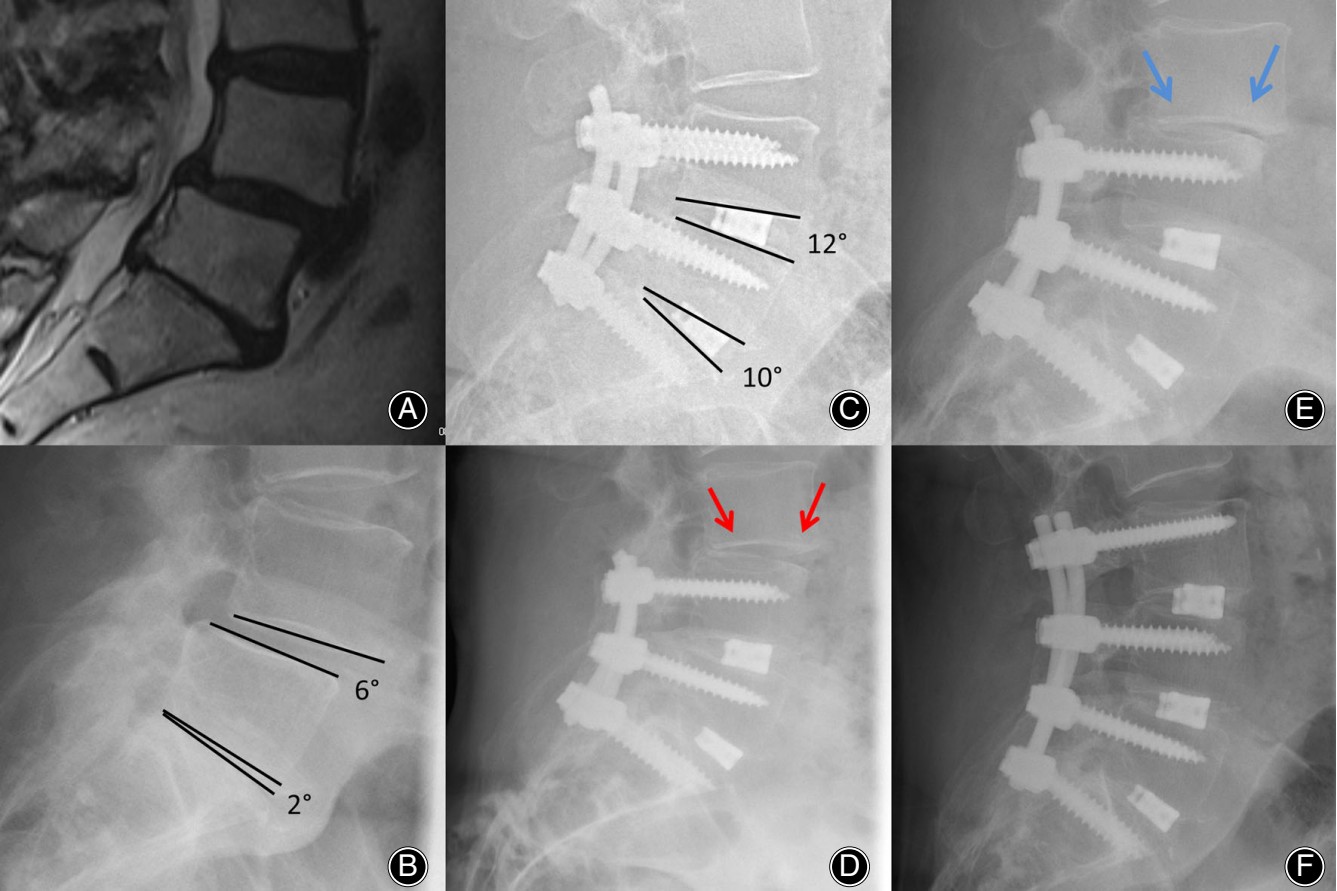
(A) Preoperative MRI scan of a 66‐year old woman with spondylolisthesis (Meyerding degree I) L4/5 and osteochondrosis L5/S1 (B) Preoperative lateral X‐ray shows a segmental lordosis L 4/5 of 6 ° and L5/S1 of 2°. (C) The follow‐up 3 months after surgery with bi‐segmental fusion and implantation of lordotic cages shows an improved segmental lordosis of 12° and 10° (increase of 6° and 8°). (D) The X‐ray control after 2 years shows a beginning adjacent disc degeneration (red arrows), which is increasing after 3 years (blue arrows, (E)) and requiring an extension of the fusion (F).

A retrospective study with a follow‐up examination was performed between July and December 2019, surgery was performed 2013 to 2016. In 2015, we switched from non‐lordotic to 5° lordotic cages in the treatment of degenerative spine diseases in our clinic. In this study, we retrospectively included 40 patients before (non‐lordotic group), and 40 patients after the switch (lordotic group, see Figs 2 and 3). Basic data (age, gender, body mass index, date of surgery, date of follow‐up, BMI, classification of indication, preoperative sacral slope) was acquired to compare the groups.

### Preoperative Evaluation

Before surgery, all study participants received a standing X‐ray in two planes and sagittal alignment parameters (LL, SL, PT) were determined. Further, an MRI scan was performed for surgical planning. A CT scan was only performed in the case of contraindications to MRI (e.g. claustrophobia or pacemaker). The VAS was recorded as a clinical parameter.

### Surgical Technique

#### Anesthesia and Position

The patients were placed in a prone position on bolsters on a radiolucent spine table after general anesthesia was administered.

#### Approach and Exposure

After a posterior midline skin incision and subperiosteally dissecting the paraspinal muscles, the laminae were exposed. The joint capsule of the cranial facet joint received special attention in order to preserve it carefully for future movements.

#### Pathological Changes and Resection

The pathologically degenerated and enlarged facet joints were resected with a chisel. The obtained bone was crushed and later deposited as spinal fusion material, as well as used for filling the cage.

#### Fixation and Placement of the Cage

**Fig. 4:**
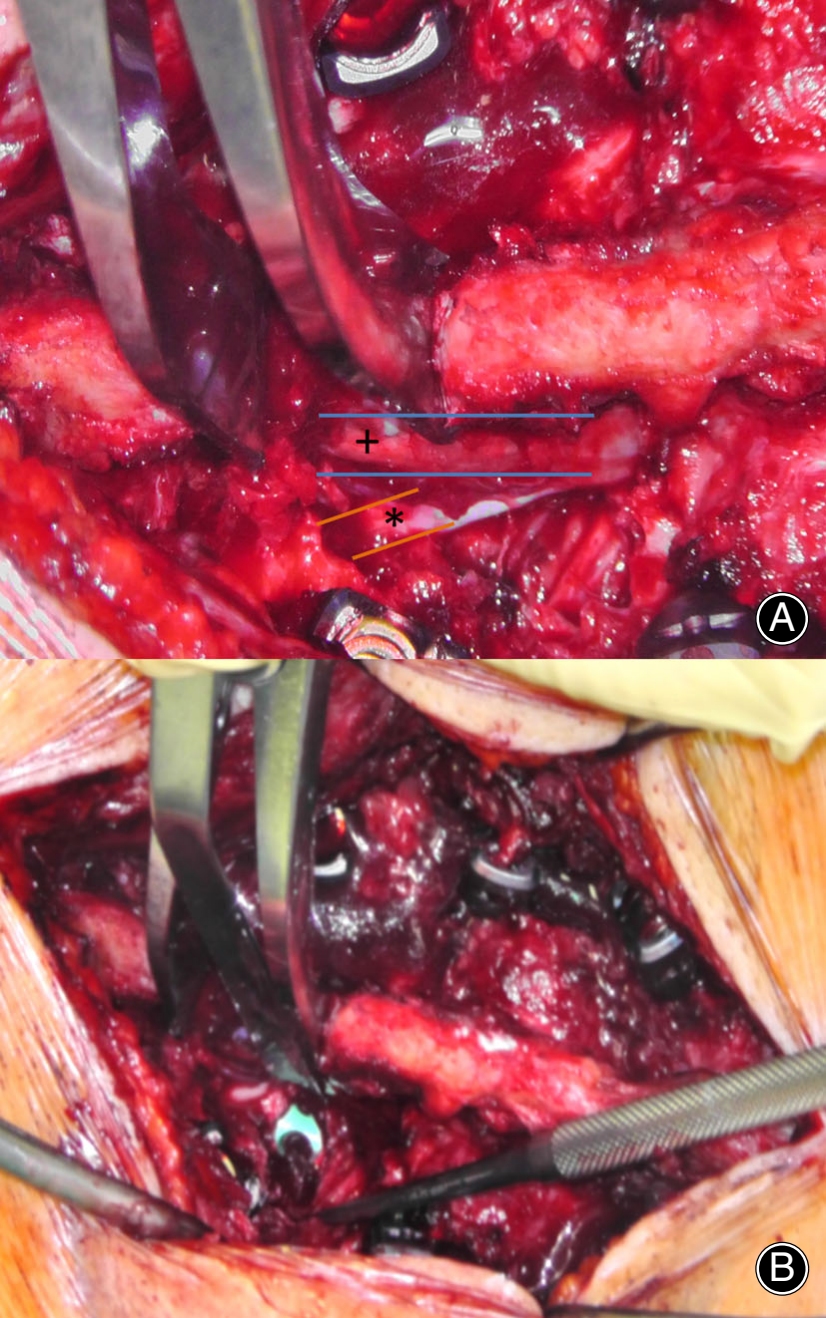
Intraoperative images. (A) Situs after exposition of the lateral part of the central dural sac (+) and the nerve root (*). (B) Situs after cage insertion (green).

Subsequently, the screws were placed using freehand technique under fluoroscopic control. Further, we resected the superior articular process with a straight osteotome to expose the lateral part of the central dural sac (see Fig. 4A), the intervertebral foramen, and the disc space. After performing a box annulotomy and a discectomy using a combination of curettes, rongeurs, and shavers, the disc space was filled with cancellous bone and the cage was inserted (see Fig. 4B).

### Reconstruction

**Fig. 5:**
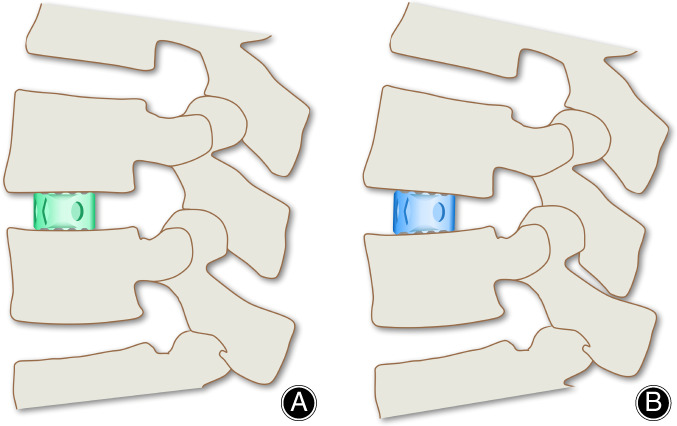
Schematic illustration of the two cage types. (A) Non‐lordotic cage resulting in lower segmental lordosis, (B) lordotic cage resulting in higher segmental lordosis.

After fluoroscopic control of the cage position, the rods were inserted. Subsequently, we compressed the screws under the rod to fix the cage and gain lordosis (see Fig. 5). Finally, the wound was closed by suture.

### Aftercare

The aftercare was carried out under pain‐adjusted full load. Before the discharge, an X‐ray of the lumbar spine in two planes was taken in a standing position. Further clinical and radiological follow‐ups were carried out after 3 and 12 months. For radiological analysis, we used the last available standing X‐ray and determined LL, SL, and PT. As part of the study, patients were invited to a clinical follow‐up in 2019.

### Outcome Measures

#### Lumbar Lordosis (LL)

**Fig. 6:**
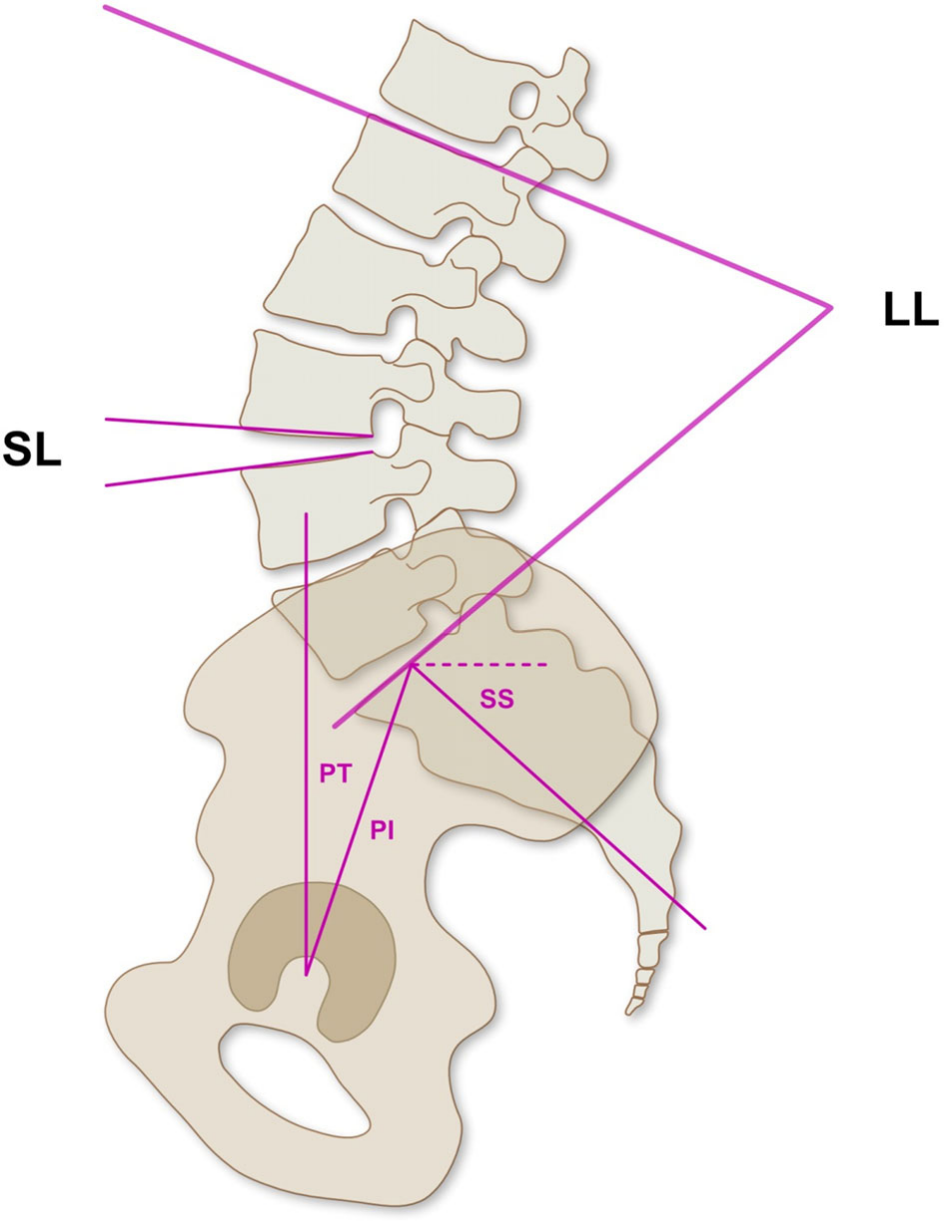
Radiographic outcome parameters. Lumbar parameters (black): lumbar lordosis (LL) and segmental lordosis (SL); spinopelvic parameters (purple): pelvic tilt (PT), sacral slope (SS) and pelvic incidence (PI).

Lumbar lordosis angle was measured using the Cobb angle method between the upper endplate of L1 and the endplate of S1 (see Fig. 6).

#### Segmental Lordosis (SL)

The segmental lordosis was measured with the Cobb angle between the endplates that are in contact with the cage (see Fig. 6).

#### Pelvic Tilt (PT)

PT was measured as the angle between the line joining the hip joint center with the midpoint of the S1 endplate and the reference vertical line (see Fig. 6).

#### Oswestry Disability Index (ODI)

Oswestry disability index (ODI) is a principal condition‐specific outcome measure used in the management of spinal disorders, to assess patient progress in routine clinical practice^26^, ^27^. The ODI score system includes 10 sections: pain intensity, personal care, lifting, walking, sitting, standing, sleeping, sex life, social life, and traveling. For each section of six statements the total score is 5. Intervening statements are scored according to rank. If more than one box is marked in each section, the highest score is taken. If all 10 sections are completed, the score is calculated as follows: total score out of total possible score × 100. If one section is missed (or not applicable) the score is calculated: (total score / (5 × number of questions answered)) × 100%. 0%–20% is considered mild dysfunction, 21%–40% is moderate dysfunction, 41%–60% is severe dysfunction, and 61%–80% is considered as disability^27^.

#### Roland–Morris Score (RMS)

The RMS is a validated form of the Roland and Morris Disability Questionnaire, which is very common in English‐speaking countries. In our study, we used the validated German translation^28^, ^29^. The RMS is available in different versions (e.g. with 16, 18, or 24 items) in which patients are asked to mark the items that apply to them and their discomfort at the time of completion, describing their back pain^30^. An item receives a score of 1, if it is checked as applicable by the respondent and a score of 0, if it is not marked. Accordingly in our study using the 24 items, total scores can vary from 0 (no disability) to 24 (severe disability). Patients with less than 9 points are considered to be less disabled and patients with more than 16 points are considered to be severely disabled.

#### Visual Analog Scale (VAS)

The VAS is a scale from 0 to 10, which the patients filled out before surgery and at follow‐up. The patient used this scale to indicate how severe or weak their pain was at the time. 0 corresponds to the best state of health and the least pain. 10 correspond to the worst state of health and most pain.

### Statistical Analysis

Data were entered in an Excel sheet (Version 14.0.7, Microsoft Office 2010, Microsoft Redmond, WA, USA) and imported into SPSS (Version 25.0.0.1, IBM, Armonk, New York, USA) for further analysis.

In order to simplify the group comparison, the difference (Δ) between the preoperative and postoperative values was calculated, being available for LL, SL, PT, and VAS. For group comparison of ODI and RMS, the values determined at follow‐up have been used. In cases with bi‐segmental fusion, we had two values for SL (upper and lower level) and therefore continued analyzing with the average of the two values.

ΔLL, ΔSL, ΔPT, and ΔVAS, ODI, and RMS were analyzed using the unpaired *t*‐test for equality of means to asses the differences between the two groups. According to the Bonferroni correction for multiple testing, the P‐value was multiplied by the number of tests (n = 6), resulting in *P*_adj_. A multivariate and univariate analysis was used to evaluate different influencing factors on postoperative ODI and RMS: cage type, gender, age, body mass index (BMI), postoperative PT and preoperative SS. We therefore categorized age (< 60 years and > 60 years), BMI (< 25; 26 to 31 and > 32), postoperative PT (< 12°, 12° to 20° and > 20°), and preoperative SS (< 35°, 35 to 45° and > 45°). The level of significance was chosen as *P* < 0.05.

## Results

### Follow‐up

The follow‐up differed on average by 0.8 years due to the study design. Due to the lack of adequate preoperative imaging, three patients of the non‐lordotic group had to be excluded. Sixty‐four of 77 patients (83.1%) were secured for a clinical follow‐up, 29 of 37 (78.4%) in the non‐lordotic and 35 of 40 (87.5%) in the lordotic group.

### General Results

The study participants suffered from degenerative diseases of the lower lumbar spine. Thirty‐four (44.2%) underwent fusion due to slight spondylolisthesis and 44 (55.8%) to osteochondrosis and spondylarthrosis; 48 (62.3%) underwent mono‐segmental fusion and 29 (37.7%) bi‐segmental fusion. Considering the basic data, we found no differences between the groups (see Table 1) in exception of the mean time of follow‐up. Regarding the treated segments, we found a maximum at L4/5 (mono‐segmental) and L4 to S1 (bi‐segmental). The distribution of treated segments did not differ significantly between the groups (see Table 2). Looking at the intraoperative findings, there are no noticeable differences between the groups. Only in some cases the introduction of the non‐lordotic cage is slightly more difficult due to the geometry.

**TABLE 1:** **B**asic data comparison of both groups

	Non‐Lordotic cage	Lordotic cage	Overall
Mean Age (SD)	60.8 (13.6)	62.7 (13.2)	61.8 (13.3)
Mean Follow‐up [years] (SD)	4.5 (0.9)	3.7 (0.9)	4.0 (1.0)
Mean BMI (SD)	28.7 (4.4)	27.1 (3.9)	27.8 (4.2)
Gender ratio [% female /% male]	43.2 / 56.8	50.0 / 50.0	46.8 / 53.2
LL preoperative [deg] (SD)	49.8 (11.5)	51.1 (11.7)	50.5 (11.6)
SL preoperative [deg] (SD)	14.1 (4.4)	13.3. (4.7)	13.7 (4.6)
PT preoperative [deg] (SD)	19.7 (6.7)	20.4 (7.7)	20.0 (7.2)
VAS preoperative (SD)	9.0 (1.2)	8.9 (1.2)	8.9 (1.2)

We observed differences only in the mean follow‐up due to the study design.

**TABLE 2:** Distribution of the treated segments in both groups

	Monosegmental				Bisegmental	
	L2/3	L3/4	L4/5	L5/S1	L3 to L5	L4 to S1
**Non‐Lordotic cage**	0 (0.0%)	2 (5.4%)	11 (29.7%)	10 (27.0%)	3 (8.1%)	11 (29.7%)
	**23 (62.2%)**				**14 (37.8%)**	
**Lordotic cage**	1 (2.5%)	1 (2.5%)	15 (37.5%)	8 (20.0%)	3 (7.5%)	12 (30.0%)
	**25 (62.5%)**				**15 (37.5%)**	
**Overall**	1 (1.3%)	3 (3.9%)	26 (33.8%)	18 (23.4%)	6 (7.8%)	23 (29.9%)
	**48 (62.3%)**				**29 (37.7%)**	

There are no relevant differences.

### Clinical Improvement

Regardless of the cage design, patients benefit significantly from surgery. A total of 93.8% (60 out of 64 patients) said in retrospect they would do surgery again and 90.6% (58 of 64 patients) were satisfied with the postoperative result. The VAS improved significantly from preoperative mean (M) = 8.9 points (Standard deviation, SD: 1.17) by 6 points to M = 2.9 (SD: 2.12, *P* < 0.001) in the follow‐up in our patient population.

### Lumbar Lordosis (LL)

**Fig. 7:**
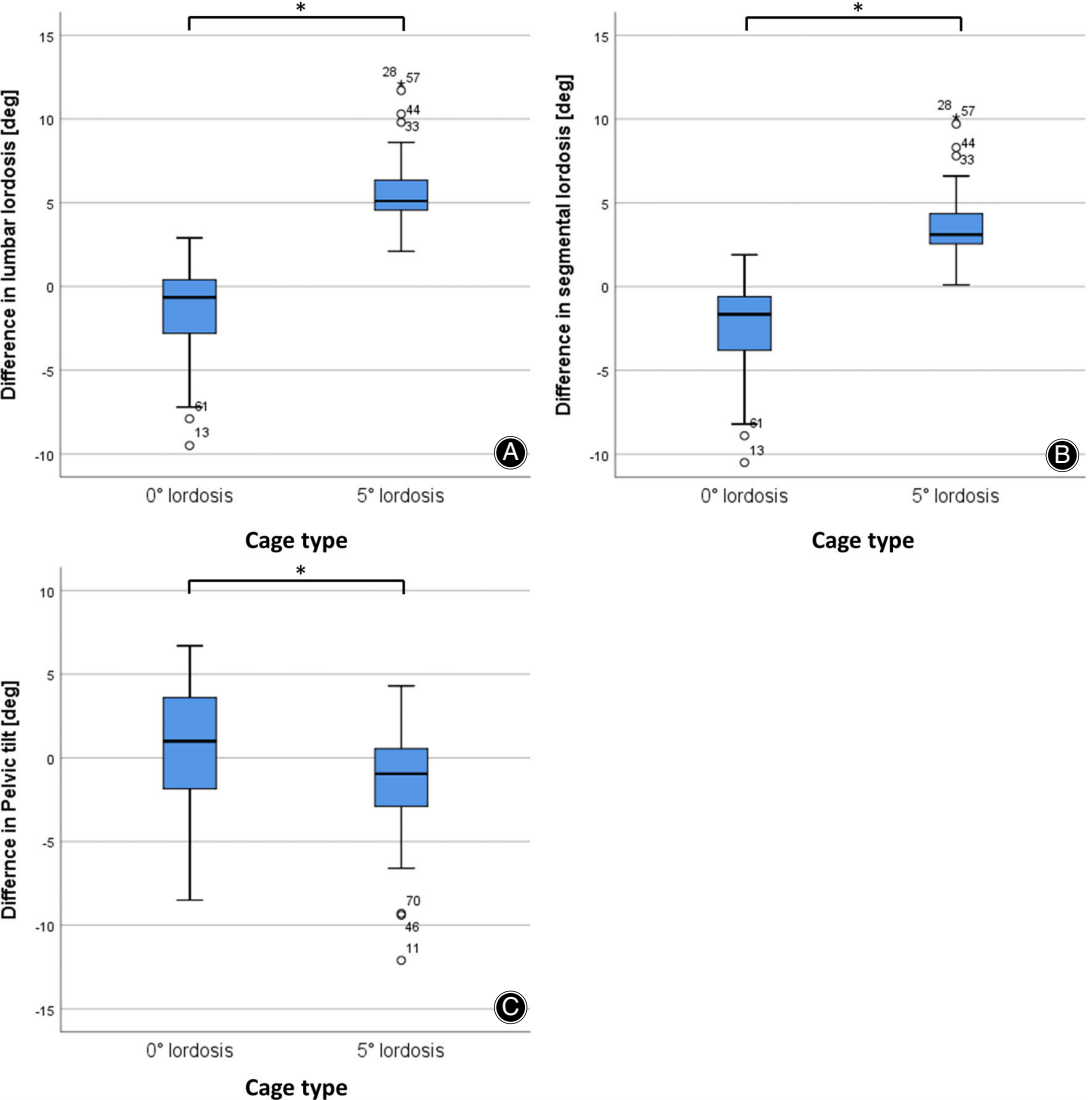
Comparison of the radiological results between the two cage types. (A) Difference in LL (preoperative *vs* postoperative) (B) segmental lordosis (C) pelvic tilt * denotes significant differences after unpaired *t*‐test and Bonferroni correction for multiple testing (*P* < 0.05).

First we asked whether we can confirm the improvement in LL, SL, and PT as described in the literature in our patient population using lordotic cages. We therefore compared ΔLL, ΔSL, ΔPT in both groups. In the case of a sample n > 30, normal distribution was assumed, despite the significant Shapiro–Wilk test (*P* < 0.05). Further, the data showed no extreme outliners (see Fig. 7) and equality of variance (Levene's test *P* > 0.05). Regarding LL, we found a decrease of 1° in NL‐group (M = 50°, SD: 11.5 preoperative to M = 49°, SD: 11.8 postoperative) and an increase of 6° in L‐group (M = 51°, SD 11.7 to M = 57°, SD: 11.7) (see Fig. 7A), resulting in a difference of 7° (13.8%) between the two groups. The unpaired *t*‐test showed a significant difference between the two cage types (*t*[73] = −8.8, *P* < 0.001, *P*_adj_ < 0.001).

### Segmental Lordosis (SL)

In SL, we found no difference in NL‐group (M = 15°, SD: 4.6 preoperative and postoperative) and increase of 5° in L‐group (M = 13°, SD = 5.0 to M = 18°, SD = 5.0) (see Fig. 7B), resulting in a difference of 5° (33.3%) between the two groups. Again, the unpaired *t*‐test offered significant differences between the two cage types (*t*[73] = −7.54, *P* < 0.001, *P*_adj_ < 0.001).

### Pelvic Tilt (PT)

In PT, we found no difference in NL‐group (M = 20°, SD 7.3 [pre] and 6.6 [post]) and a decrease of 2° in L‐group (M = 21°, SD 7.6 to M = 19°, SD: 7.2) (see Fig. 7 c) resulting in a difference of 2° (10.0%) between the two groups. Regarding the *t*‐test, we found small but significant differences between the two cage types (*t*[73] = 2.71, *P* = 0.008, *P*_adj_ = 0.048).

In the synopsis, the expected radiological improvements in LL, SL, and PT can be confirmed, measured on the standing X‐ray.

### Oswestry Disability Index (ODI)

**Fig. 8:**
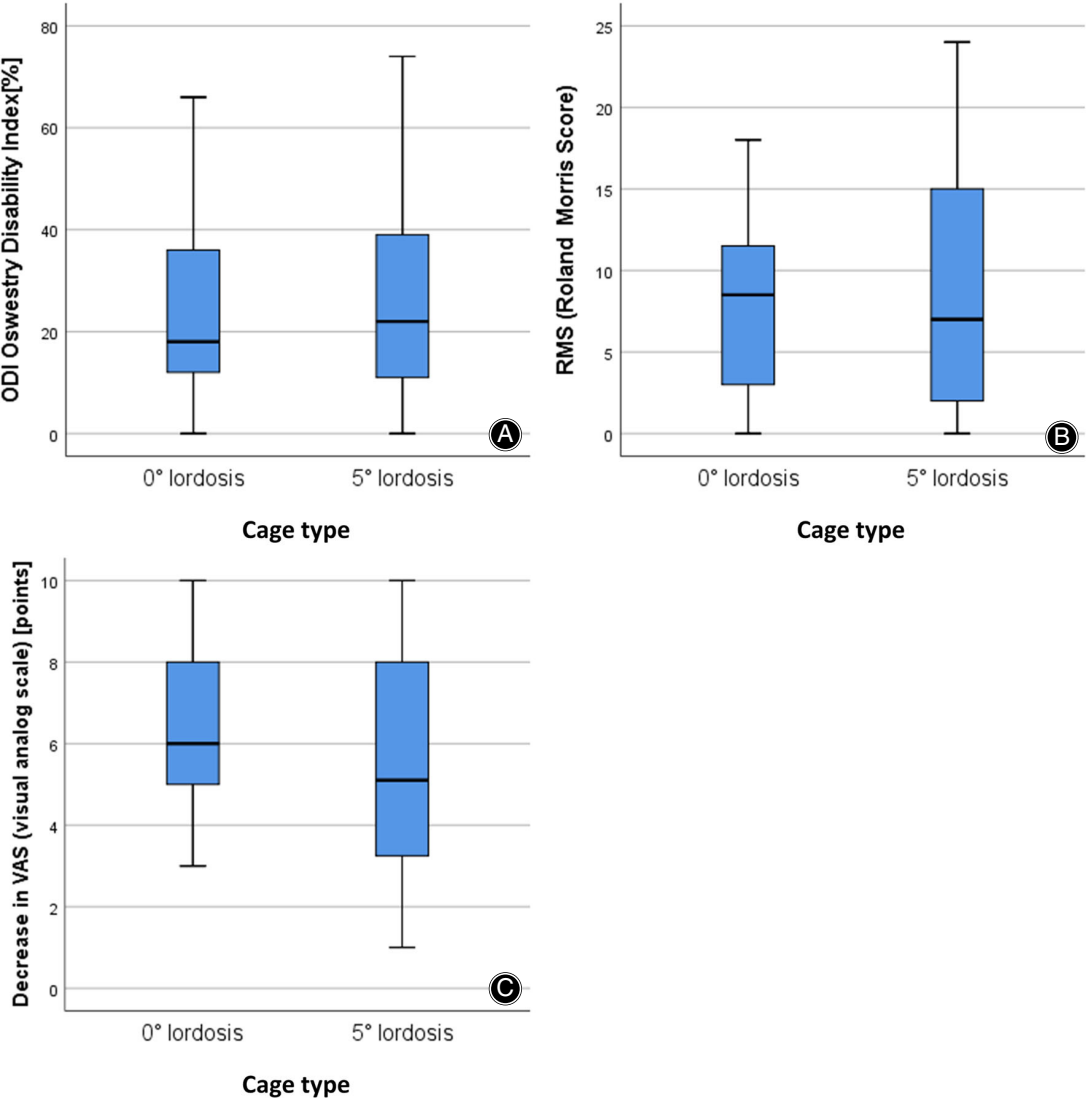
Comparison of the clinical results between the two cage types. (A) ODI (postoperative) (B) Roland–Morris Score (postoperative) (C) Difference in VAS (preoperative *vs* postoperative). All clinical scores show no significant differences between the two cage types.

Second, we wanted to investigate if the radiological improvement leads to a superior clinical outcome using lordotic cages compared with non‐lordotic cages in the follow‐up. We therefore compared ODI, RMS, and ΔVAS. Regarding the ODI at follow‐up, we found a mean of 25.5% (SD: 19.5) according to a moderate disability in the whole population. In group comparison, slightly higher values were found in lordotic cage group (M = 28.3%, SD: 21.4) compared with non‐lordotic cage group (M = 22.6, SD: 16.7, 5.7% difference) without significant differences in *t*‐test (*t*[62] = −1.16, *P* = 0.25, *P*_adj_ > 0.99) (see Fig. 8A).

### Roland–Morris Score (RMS)

The mean RMS at follow up was 8.1 (SD: 6.4), according to a small disability. In group comparison, we could distinguish similar results in both groups (lordotic cage: M = 9 points, SD: 7.2 *vs* non‐lordotic cage (M = 8 points, SD 5.3, 1 point difference) without significant differences in t‐test (*t*[62] = −0.78, *P* = 0.48, *P*_adj_ > 0.99) (see Fig. 8B).

### Visual Analog Scale (VAS)

In group comparison, VAS shows a slightly higher starting point (VAS 9.0, SD 1.12)) in the non‐lordotic group compared to the lordotic group (VAS 8.9, SD 1.12). The end point is somewhat lower in the non‐lordotic group (VAS 2.6, SD 1.81) compared to the lordotic group (VAS 3.4, SD 2.31), so that in the summary the difference preoperatively to postoperatively is minimally higher in the non‐lordotic group (6.4 points decrease *vs* 5.5 points decrease, 0.9 points difference) without statistical significance in the unpaired t‐test (*t*[62] = 1.54, *P* = 0.128, *P*_adj_ = 0.768) (see Fig. 8C).

### Subgroup Analysis

To enable a deeper understanding of the data set, we calculated the differences in ODI, RMS, and VAS between the cage groups for sex, age, follow‐up time, classification of indication, and BMI (see Table 3). Significant better results for the NL‐Group in VAS were found in osteochondrosis (classification of indication) (M = 6.7 *vs* 5.2; Δ = 1.5, *P* = 0.029) and for follow‐up time less than 4 years (M = 6.6 *vs* 4.7; Δ = 1.4; *P* = 0.047). The L‐group showed significantly better results in follow‐up time of more than 4 years (M = 6.4 *vs* 8.2; Δ = 1.8; *P* = 0.035). In summary, in the long‐term follow‐up of more than 4 years, there appears to be a tendency towards the clinical superiority of the lordotic cage. However, this subgroup (lordotic cage and more than 4 years follow‐up) is rather small with eight patients.

**TABLE 3:** Subgroup analysis for age, gender, classification, follow‐up time, and BMI

	Number of patients (N)		ODI		RMS		ΔVAS	
Characteristics	NL‐Group	L‐Group	NL‐Group	L‐Group	NL‐Group	L‐Group	NL‐Group	L‐Group
*Sex*								
female	13	18	13.4 (SD: 10.5)	21.7 (SD: 19.3)	5.6 (SD: 5.0)	5.9 (SD: 6.7)	6.2 (SD:2.4)	6.5 (SD:2.5)
male	16	17	30.1 (SD: 17.6)	35.2 (SD: 21.9)	9.1 (SD: 5.2)	11.5 (SD: 6.7)	6.6 (SD: 1.5)	4.5 (SD: 2.3)
*Age*								
≤ 60 years	15	14	20.3 (SD: 16.3)	25.1 (SD 20.7)	6.5 (SD: 5.3)	7.2 (SD:7.1)	6.8 (SD:2.0)	6.0 (SD:2.5)
> 60 years	14	21	25.1 (SD: 17.8)	30.4 (SD:22.2)	8.5 (SD: 5.3)	9.6 (SD: 7.2)	6.0 (SD: 1.7)	5.2 (SD: 2.7)
*Follow‐up time*								
≤ 4 years	8	27	25.0 (SD: 20.4)	31.7 (SD: 21.6)	8.3 (SD: 5.9)	9.4 (SD: 7.2)	6.6 (SD: 1.3)*	4.7 (SD: 2.4)*
> 4 years	21	8	21.7 (SD: 15.8)	16.8 (SD: 17.3)	7.2 (SD: 5.2)	6.0 (SD: 6.9)	6.4 (SD: 2.1)*	8.2 (SD: 1.4)*
*Classification*								
Osteochondrosis	20	18	23.6 (SD: 18.7)	26.4 (SD: 19.0)	8.2(SD: 5.9)	9.1(SD: 6.8)	6.7(SD: 2.0)*	5.2(SD: 2.1)*
Spondylolisthesis	9	17	20.4 (SD: 12.4)	30.2 (SD: 24.1)	6.1 (SD: 3.5)	8.2 (SD: 7.8)	5.8 (SD: 1.6)	5.9 (SD: 3.1)
*BMI*								
≤ 25	7	12	14.0 (SD: 10.5)	14.5 (SD: 9.9)	3.9 (SD: 3.6)	4.5 (SD: 3.5)	6.9 (SD: 2.0)	6.5 (SD: 2.2)
25 to 31	11	10	22.5 (SD: 15.3)	31.6 (SD: 20.8)	8.5 (SD: 4.5)	9.9 (SD: 7.5)	6.3 (SD: 2.0)	6.2 (SD: 2.9)
> 32	7	5	32.3 (SD: 22.8)	50.0 (SD: 25.7)	10.6 (SD: 6.8)	14.0 (SD: 8.5)	5.4 (SD: 1.5)	4.4 (SD: 2.2)

* Denotes significant differences in group comparison (*P* < 0.05).

In conclusion, we had to reject our hypothesis that the radiological improvement leads to a superior clinical outcome using lordotic cages in the follow‐up.

### Independent Influence Factors

#### Multivariate Analysis

Further, we searched for influencing factors on ODI and RMS. Therefore. a multivariate analysis (MANOVA) was used to evaluate different factors (BMI, age, gender, cage type, and preoperative SS) on postoperative ODI and RMS. The results indicate a significant multivariate influence across all variables of BMI (Wilks λ = 0.57, F [4, 44] = 3.61, *P* = 0.012) and preoperative SS (Wilks λ = 0.66, F [4, 44] = 2.54, *P* = 0.048). Age, gender, cage type, and postoperative PT had no significant influence (*P* > 0.05).

#### Univariate Analysis

Regarding the univariate analysis, we found again a significant influence of BMI on ODI (F [2, 23] = 6.08, *P* = 0.008) and on RMS (F [2, 23] = 7.21, *P* = 0.004).

In conclusion, we were able to proclaim BMI and preoperative SS as clear influencing factors on the clinical outcome.

### Implants Evaluation

In the follow‐up, we found no pseudoarthrosis or implant associated complications like screw loosening, cage dislocation, or rod breakage. Metal removal due to allergic reactions was not necessary in our population.

### Complications

**Fig. 9:**
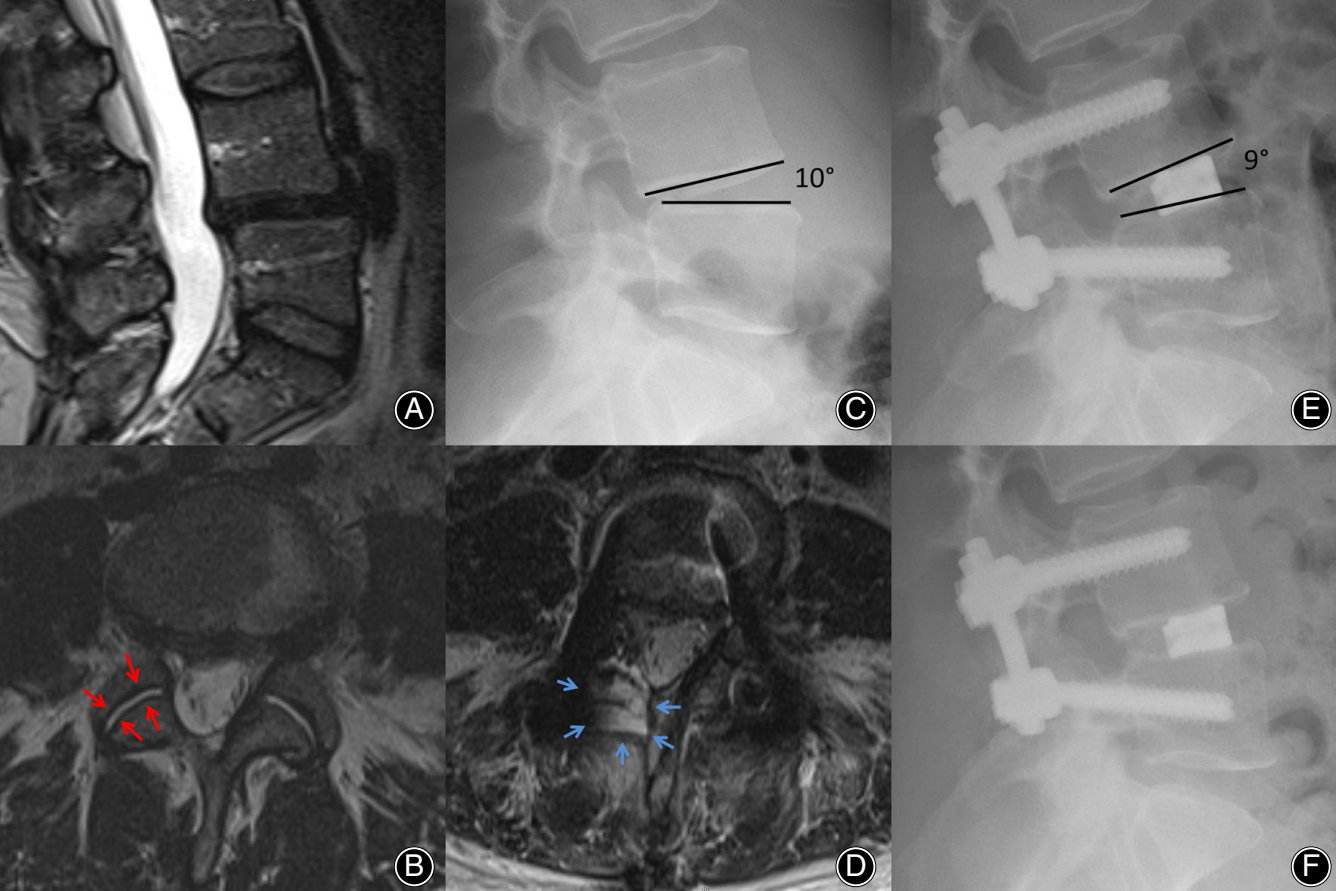
(A) Preoperative MRI scan of a 70‐year old man with an instability of L4/5 after previous decompression surgery. (B) Transverse view (MRI) at level L 4/5 with fluid sign (red arrows) as a sign of instability (C) The preoperative radiograph in a standing position shows segmental hyperlordosis of 10° and a retrolisthesis. (D) Three days after surgery the transverse view (MRI) demonstrates an accumulation of liquor after suturing of the dura (blue arrows). (E) The lateral X‐ray shows a slight decrease in segmental lordosis by 1° to 9°. (F) In the follow‐up after 2 years, a secure bony fusion is evident.

As complications, we had three intraoperative dura injuries treated with suture and Tachosil® patch (matrix with human fibrinogen and human thrombin) (see Fig. 9). All dura injuries were noticed intraoperatively and healed without consequences. Two patients had to be revised superficially due to wound‐healing disorders.

## Discussion

In this study, we report an enormous increase in LL, SL and a small increase in PT using a lordotic cage *vs* very small improvement in non‐lordotic cages, resulting in a clear, significant difference between the two groups. Further, we could not confirm a superior clinical outcome using lordotic cages compared with non‐lordotic cages in the follow‐up, but could identify BMI and preoperative SS as clear influencing factors on clinical outcome.

### Cage Design and Approach

The use of a TLIF cage in degenerative spine surgery of the lumbar spine has become popular in recent decades due to many advantages, but with anterior lumbar interbody fusion (ALIF) and the extreme lateral interbody fusion (XLIF) approach there are also alternatives.

Comparing TLIF with ALIF^10^, which also requires an anterior approach, there is a shorter mean OR time, less blood loss, less patients requiring ICU, and a shorter hospital stay in TLIF procedure^31^. In biomechanical studies, the TLIF cage in combination with pedicle screws shows comparable segmental stability to the ALIF cage, despite the smaller contact area^17^.

Another alternative to the TLIF is the XLIF, which was first published in 2006^19^. Contrasting the two techniques, Sembrano *et al*. found similar clinical outcomes after 2 years^32^. TLIF group had superior improvement of ODI (57%) compared to XLIF (53%) and lower complication rate, so hip flexion weakness only occurred in the XLIF group (31%). However, XLIF group had lower blood loss (79% of cases <100 mL) compared to TLIF group. Based on these facts, we still see the TLIF technique used in this study as the leading interbody fusion technique for majority of the cases.

### Radiological Results

Improving the LL is an undisputed goal in lumbar spine surgery and had a major impact on the development of cages in recent years. Examining the effect of lordotic cages, Gödde *et al*. observed that lordotic cages significantly increase SL (3–8°) and LL (45° to 53°) using posterior interbody fusion technique (PLIF)^20^. We found comparable results with an improvement of 5° in SL and 6° in LL using lordotic cages. Sembrano *et al*. also reproduced the significant increase in SL using lordotic XLIF cages compared to non‐lordotic XLIF cages but to a smaller extent (2.8° to 0.6°)^21^. This small increase is surprising, especially since in the XLIF technique, a direct expansion of the intervertebral disc compartment is possible. Other studies found higher increases in SL using XLIF cages. So, Malham *et al*. found a nearly doubled increase of 7.9°–9.4° in SL using XLIF cages^33^. Juxtaposing ALIF, XLIF, and TLIF Watkins *et al*. could prove the highest increase in SL (4.5°) in ALIF procedure, due to the possibility to resect the anterior longitudinal ligament, followed by XLIF procedure (2.2°), and TLIF procedure (0.8°).

In conclusion, ALIF and XLIF cages seem to be a better possibility for relordosing the lumbar spine compared to TLIF cages, despite the wide range of values. However, our data show a higher correcting potential of the TLIF cage compared to other studies.

Contemplating the clinical outcome of lumbar interbody fusion techniques, the literature shows good postoperative functional scores. So, Schimmel *et al*. indicate a mean postoperative ODI score of 31% in lumbar interbody fusion using PEEK cages and an anterior approach^6^. Observing one‐level fusions with TLIF cags, Hackenberg *et al*. found an mean postoperative ODI of 33%^34^. In our study we found a lower mean postoperative ODI with 25.5% using posterior open approach and TLIF cages. However, there are also studies in the literature with even lower postoperative ODI values, such as Park *et al*. with 16% ODI 2 years postoperatively after minimally invasive TLIF or Pavlov *et al*. with 24% after ALIF^10^, ^35^. In summary, no superior interbody fusion technique can be found, especially since the postoperative ODI scores vary widely depending on the patient population.

### Clinical Results

The focus of our work was the relationship between sagittal alignment parameters and postoperative pain. Previous studies were able to prove the interrelation between LL and lumbar back pain. In an epidemiological study by Tsuji *et al*. including 509 people aged 50–85 years, VAS was significantly inversely correlated with LL^36^. However, it must be mentioned that the difference in LL between the groups in this study was only 4°. This result is supported by a large meta‐analysis with 796 patients with LBP and 927 healthy controls. Here, Chun *et al*. revealed a strong relationship between LBP and decreased LL^37^. Also Schwab *et al*. could document a clear correlation of LL and ODI in adult deformity^38^. Due to the relation between LBP and decreased LL, we hypothesized that a postoperative increase in LL also leads to an improvement in clinical outcome, but we could not confirm a correlation.

From the authors' perspective, the following reasons are conceivable. First, in large cases with pedicle subtraction osteotomies and an enormous change in the sagittal profile, an influence of the LL (especially in relation to the pelvic incidence) on the clinical outcome has been proven^39^. However, to the best of our knowledge, no study on mono‐ or bi‐segmental fusion was able to demonstrate a connection between improvement in LL and improvement in VAS. Therefore, in the present study, with a special perspective on the cage design, a lack of correlation between LL and VAS is not surprising. Secondly, the effect described above seems to be relatively small and may only be detectable in very large populations. For example, other studies like Ashraf *et al*. found no significant correlation between LL and ODI, in 150 patients with lumbar back pain^40^. Finally, the influence of LL might be overlaid by other causes of postoperative pain, such as residual lumbosacral pain referring to the sacroiliac joints or the sacrosciatic ligaments and scarred changes in the paraspinal muscles, unlike in populations without previous surgery^41^.

### Independent Influencing Factors on Functional Outcome

Other interesting parameters are the sagittal alignment angles PT and SS. PT is often increased in patients with degenerative changes in the lumbar spine (pelvic retroversion) to compensate a decreased LL. In the literature, some authors were able to confirm a correlation between PT and clinical outcome after spinal fusion. The normal value for PT is 12° (SD: 7.0), according to an investigation of 268 asymptomatic Caucasian and Japanese subjects^42^. Based on this study, significantly increased values with over 20° PT could be found in our population. But elevated postoperative values for PT after lumbar fusion are previously described in literature (Lazennec: 22.2°, Bourghli: 19.9°)^43^, ^44^. Considering the multivariate analysis, no correlation of postoperative PT and clinical outcome could be concluded in our data, as other authors have done for patients with spondylolisthesis and lumbosacral fusion^45^. However, in the first publication dealing with a rather small number of patients, many different radiological and clinical parameters were correlated, so the problem of multiple testing may not have been sufficiently taken into account. The second publication argues with two groups: The first group without postoperative pain had a postoperative PT of 14°, the second group with postoperative pain had a PT of 26°, which differs significantly. In the second group, however, the PT increased due to fusion with an average of 6° (20° to 26°), which leads to a significant deterioration in sagittal alignment and understandably leads to an increase in pain. In summary, no study can be found that can prove a connection between PT and VAS in short‐segment fusions.

Regarding SS, we have categorized the data according to the Roussouly classification^46^. In his classification, Roussouly describes type 4 with SS over 45 ° and an increased tendency to spondylarthrosis and spinal stenosis. In our data, patients with preoperative SS over 45° (N = 8) postoperatively showed a worse outcome with on average almost twice as high ODI values compared to the other patients. We therefore recommend giving SS special consideration in preoperative planning and indication.

### Study Limitations

Due to the study design, the time to follow‐up is different in both groups. This may lead to some bias, particularly regarding the clinical outcome. However, the follow‐up period is quite long so this difference should not have a major impact. In order to eliminate this problem, a prospective study should be carried out.

### Conclusion

In this study, we were able to show that the correction potential of lordotic TLIF cages is comparable or, depending on the study, even superior to anterolateral inserted cages. We therefore recommend using lordotic cages. Referring to the sagittal profile, it is generally accepted that upright standing means the need to maintain balance. This requires an intact profile of the spine in harmonious conjunction with ligamentous and muscular structures. Nevertheless, neither in this study with the main focus on the cage design nor other comparable studies has it been possible to prove the clinical benefit of improved sagittal alignment. Finally, we think that factors such as the correct indication, adequate decompression, and careful handling of the soft tissues make up the main part of the success, and the minimal difference in the LL is therefore of subordinate importance.

#### Authorship Declaration

All authors listed meet the authorship criteria according to the latest guidelines of the International Committee of Medical Journal Editors and all authors are in agreement with the manuscript.

## Authors Contributions Statement

CW did the surgery, compiled the statistics and wrote the manuscript. TB helped with the study design and did the follow‐up examination. SF did the statistics and helped with the study design. LE did the radiological analysis and help with the statistics. MM developed the study design and co‐wrote the manuscript. All authors have read and approved the final submitted manuscript.

## Availability of Data and Material

The datasets used and/or analyzed during the current study are available from the corresponding author on reasonable request.

## References

[1] Stovall DO Jr , Goodrich JA , Lundy D , Standard SC , Joe C , Preston CD . Sacral fixation technique in lumbosacral fusion. Spine, 1997, 22: 32–37. 9122779 10.1097/00007632-199701010-00006

[2] Yoshihara H . Surgical options for lumbosacral fusion: biomechanical stability, advantage, disadvantage and affecting factors in selecting options. Eur J Orthop Surg Traumatol, 2014, 24: 73–82. 23860810 10.1007/s00590-013-1282-2

[3] Polly DW Jr , Klemme WR , Cunningham BW , Burnette JB , Haggerty CJ , Oda I . The biomechanical significance of anterior column support in a simulated single‐level spinal fusion. J Spinal Disord., 2000, 13: 58–62. 10710152 10.1097/00002517-200002000-00012

[4] Ray CD . Threaded titanium cages for lumbar interbody fusions. Spine., 1997, 22: 667–680. 9089940 10.1097/00007632-199703150-00019

[5] Rapoff AJ , Ghanayem AJ , Zdeblick TA . Biomechanical comparison of posterior lumbar interbody fusion cages. Spine., 1997, 22: 2375–2379. 9355218 10.1097/00007632-199710150-00010

[6] Schimmel JJ , Poeschmann MS , Horsting PP , Schonfeld DH , van Limbeek J , Pavlov PW . PEEK cages in lumbar fusion: mid‐term clinical outcome and radiologic fusion. Clin Spine Surg., 2016, 29: 252–258. 10.1097/BSD.0b013e31826eaf74 27196005

[7] Lee CK , Langrana NA . Lumbosacral spinal fusion. A biomechanical study. Spine., 1984, 9: 574–581. 6495027 10.1097/00007632-198409000-00007

[8] Lin PM , Cautilli RA , Joyce MF . Posterior lumbar interbody fusion. Clin Orthop Relat Res., 1983, 180: 154–168. 6354543

[9] Zhang Z , Li H , Fogel GR , Liao Z , Li Y , Liu W . Biomechanical analysis of porous additive manufactured cages for lateral lumbar Interbody fusion: a finite element analysis. World Neurosurg., 2018, 111: 581–591. 10.1016/j.wneu.2017.12.127 29288855

[10] Pavlov PW , Meijers H , van Limbeek J , *et al*. Good outcome and restoration of lordosis after anterior lumbar interbody fusion with additional posterior fixation. Spine., 2004, 29: 1893–1900. 15534411 10.1097/01.brs.0000137067.68630.70

[11] Cloward RB . Posterior lumbar interbody fusion updated. Clin Orthopaed Related Res., 1985, 193: 16–19. 3971616

[12] Goh S , Tan C , Price RI , *et al*. Influence of age and gender on thoracic vertebral body shape and disc degeneration: an MR investigation of 169 cases. J Anat., 2000, 197: 647–657. 11197538 10.1046/j.1469-7580.2000.19740647.x PMC1468180

[13] Harms JG , Jeszenszky D . Die posteriore, lumbale, interkorporelle Fusion in unilateraler transforaminaler Technik. Operative Orthopädie Und Traumatologie., 1998, 10: 90–102. 17332991 10.1007/s00064-006-0112-7

[14] Moskowitz A . Transforaminal lumbar interbody fusion. Orthop Clin North Am., 2002, 33: 359–366. 12389281 10.1016/s0030-5898(01)00008-6

[15] Zhang Q , Yuan Z , Zhou M , Liu H , Xu Y , Ren Y . A comparison of posterior lumbar interbody fusion and transforaminal lumbar interbody fusion: a literature review and meta‐analysis. BMC Musculoskelet Disord., 2014, 15: 367. 25373605 10.1186/1471-2474-15-367 PMC4232693

[16] Rosenberg WS , Mummaneni PV . Transforaminal lumbar interbody fusion: technique, complications, and early results. Neurosurgery., 2001, 48: 569–574. 11270547 10.1097/00006123-200103000-00022

[17] Niemeyer TK , Koriller M , Claes L , Kettler A , Werner K , Wilke HJ . In vitro study of biomechanical behavior of anterior and transforaminal lumbar interbody instrumentation techniques. Neurosurgery., 2006, 59: 1271–1277. 17277690 10.1227/01.NEU.0000245609.01732.E4

[18] Regan JJ , Aronoff RJ , Ohnmeiss DD , Sengupta DK . Laparoscopic approach to L4–L5 for Interbody fusion using BAK cages: experience in the first 58 cases. Spine., 1999, 24: 2171–2174. 10543017 10.1097/00007632-199910150-00018

[19] Ozgur BM , Aryan HE , Pimenta L , Taylor WR . Extreme lateral Interbody fusion (XLIF): a novel surgical technique for anterior lumbar interbody fusion. Spine J., 2006, 6: 435–443. 16825052 10.1016/j.spinee.2005.08.012

[20] Godde S , Fritsch E , Dienst M , Kohn D . Influence of cage geometry on sagittal alignment in instrumented posterior lumbar interbody fusion. Spine., 2003, 28: 1693–1699. 12897494 10.1097/01.BRS.0000083167.78853.D5

[21] Sembrano JN , Horazdovsky RD , Sharma AK , Yson SC , Santos ERG , Polly DW Jr . Do Lordotic cages provide better segmental Lordosis versus Nonlordotic cages in lateral lumbar Interbody fusion (LLIF)? Clin Spine Surg., 2017, 30: 338–343. 10.1097/BSD.0000000000000114 28437335

[22] Gelb DE , Lenke LG , Bridwell KH , Blanke K , McEnery KW . An analysis of sagittal spinal alignment in 100 asymptomatic middle and older aged volunteers. Spine., 1995, 20: 1351–1358. 7676332

[23] Vialle R , Levassor N , Rillardon L , Templier A , Skalli W , Guigui P . Radiographic analysis of the sagittal alignment and balance of the spine in asymptomatic subjects. J Bone Joint Surg Am., 2005, 87: 260–267. 15687145 10.2106/JBJS.D.02043

[24] Carlson BB , Saville P , Dowdell J , *et al*. Restoration of lumbar lordosis after minimally invasive transforaminal lumbar interbody fusion: a systematic review. Spine J., 2019, 19: 951–958. 30529420 10.1016/j.spinee.2018.10.017

[25] Been E , Kalichman L . Lumbar lordosis. Spine J., 2014, 14: 87–97. 24095099 10.1016/j.spinee.2013.07.464

[26] Roland M , Morris R . A study of the natural history of back pain. Part I: development of a reliable and sensitive measure of disability in low‐back pain. Spine., 1983, 8: 141–144. 6222486 10.1097/00007632-198303000-00004

[27] Mannion AF , Junge A , Fairbank JC , Dvorak J , Grob D . Development of a German version of the Oswestry disability index. Part 1: cross‐cultural adaptation, reliability, and validity. Eur Spine J., 2006, 15: 55–65. 15856341 10.1007/s00586-004-0815-0 PMC3454571

[28] Wiesinger GF , Nuhr M , Quittan M , Ebenbichler G , Wolfl G , Fialka‐Moser V . Cross‐cultural adaptation of the Roland‐Morris questionnaire for German‐speaking patients with low back pain. Spine., 1999, 24: 1099–1103. 10361659 10.1097/00007632-199906010-00009

[29] Exner V , Keel P . Measuring disability of patients with low‐back pain—validation of a German version of the Roland & Morris disability questionnaire. Schmerz., 2000, 14: 392–400. 12800012 10.1007/s004820000010

[30] Stratford PW , Binkley JM . Measurement properties of the RM‐18: a modified version of the Roland‐Morris disability scale. Spine., 1997, 22: 2416–2421. 9355224 10.1097/00007632-199710150-00018

[31] Hee HT , Castro FP Jr , Majd ME , Holt RT , Myers L . Anterior/posterior lumbar fusion versus transforaminal lumbar interbody fusion: analysis of complications and predictive factors. J Spinal Disord., 2001, 14: 533–540. 11723406 10.1097/00002517-200112000-00013

[32] Sembrano JN , Tohmeh A , Isaacs R , Group SDS . Two‐year comparative outcomes of MIS lateral and MIS Transforaminal Interbody fusion in the treatment of degenerative Spondylolisthesis: part I: clinical findings. Spine., 2016, 41: 123–132. 10.1097/BRS.0000000000001471 26825788

[33] Malham GM , Ellis NJ , Parker RM , *et al*. Maintenance of segmental Lordosis and disk height in stand‐alone and instrumented extreme lateral Interbody fusion (XLIF). Clin Spine Surg., 2017, 30: 90–98. 10.1097/BSD.0b013e3182aa4c94 28207620

[34] Hackenberg L , Halm H , Bullmann V , Vieth V , Schneider M , Liljenqvist U . Transforaminal lumbar interbody fusion: a safe technique with satisfactory three to five year results. Eur Spine J., 2005, 14: 551–558. 15672243 10.1007/s00586-004-0830-1 PMC3489237

[35] Park P , Foley KT . Minimally invasive transforaminal lumbar interbody fusion with reduction of spondylolisthesis: technique and outcomes after a minimum of 2 years' follow‐up. Neurosurg Focus., 2008, 25: 16. 10.3171/FOC/2008/25/8/E16 18673045

[36] Tsuji T , Matsuyama Y , Sato K , Hasegawa Y , Yimin Y , Iwata H . Epidemiology of low back pain in the elderly: correlation with lumbar lordosis. J Orthop Sci., 2001, 6: 307–311. 11479757 10.1007/s007760100023

[37] Chun SW , Lim CY , Kim K , Hwang J , Chung SG . The relationships between low back pain and lumbar lordosis: a systematic review and meta‐analysis. Spine J., 2017, 17: 1180–1191. 28476690 10.1016/j.spinee.2017.04.034

[38] Schwab F , Farcy JP , Bridwell K , *et al*. A clinical impact classification of scoliosis in the adult. Spine., 2006, 31: 2109–2114. 16915098 10.1097/01.brs.0000231725.38943.ab

[39] Schwab FJ , Patel A , Shaffrey CI , *et al*. Sagittal realignment failures following pedicle subtraction osteotomy surgery: are we doing enough? J Neurosurg Spine., 2012, 16: 539. 22462571 10.3171/2012.2.SPINE11120

[40] Ashraf A , Farahangiz S , Pakniat Jahromi B , Setayeshpour N , Naseri M , Nasseri A . Correlation between radiologic sign of lumbar Lordosis and functional status in patients with chronic mechanical low Back pain. Asian Spine J., 2014, 8: 565–570. 25346808 10.4184/asj.2014.8.5.565 PMC4206805

[41] Schwarzer AC , Aprill CN , Bogduk N . The sacroiliac joint in chronic low Back pain. Spine., 1995, 20: 31–37. 7709277 10.1097/00007632-199501000-00007

[42] Le Huec JC , Hasegawa K . Normative values for the spine shape parameters using 3D standing analysis from a database of 268 asymptomatic Caucasian and Japanese subjects. Eur Spine J., 2016, 25: 3630–3637. 26951168 10.1007/s00586-016-4485-5

[43] Lazennec JY , Ramare S , Arafati N , *et al*. Sagittal alignment in lumbosacral fusion: relations between radiological parameters and pain. Eur Spine J., 2000, 9: 47–55. 10766077 10.1007/s005860050008 PMC3611353

[44] Bourghli A , Aunoble S , Reebye O , Le Huec JC . Correlation of clinical outcome and spinopelvic sagittal alignment after surgical treatment of low‐grade isthmic spondylolisthesis. Eur Spine J., 2011, 20: 663–668. 21809014 10.1007/s00586-011-1934-z PMC3175926

[45] Ren H , Geng W , Ma J , *et al*. Correlation analysis of changes of spine‐pelvic sagittal parameters before and after operation and effectiveness in patients with lumbar Spondylolisthesis. Zhongguo Xiu Fu Chong Jian Wai Ke Za Zhi., 2015, 29: 1269–1274. 26749737

[46] Roussouly P , Gollogly S , Berthonnaud E , Dimnet J . Classification of the normal variation in the sagittal alignment of the human lumbar spine and pelvis in the standing position. Spine., 2005, 30: 346–353. 15682018 10.1097/01.brs.0000152379.54463.65

